# Identification of Biomarkers for Controlling Cancer Stem Cell Characteristics in Bladder Cancer by Network Analysis of Transcriptome Data Stemness Indices

**DOI:** 10.3389/fonc.2019.00613

**Published:** 2019-07-04

**Authors:** Shen Pan, Yunhong Zhan, Xiaonan Chen, Bin Wu, Bitian Liu

**Affiliations:** ^1^Department of Radiology, Shengjing Hospital of China Medical University, Shenyang, China; ^2^Department of Urology, Shengjing Hospital of China Medical University, Shenyang, China

**Keywords:** bladder cancer, cancer cell stemness, WGCNA, mRNAsi, TCGA, biomarker

## Abstract

**Background:** Stem cells characterized by self-renewal and therapeutic resistance play crucial roles in bladder cancer (BLCA). However, the genes modulating the maintenance and proliferation of BLCA stem cells are still unclear. In this study, we aimed to characterize the expression of stem cell-related genes in BLCA.

**Methods:** The mRNA expression-based stemness index (mRNAsi) of The Cancer Genome Atlas (TCGA) was evaluated and corrected by tumor purity. Corrected mRNAsi were further analyzed with regard to muscle-invasive bladder cancer molecular subtypes, survival analysis, pathological staging characteristics, and outcomes after primary treatment. Next, weighted gene co-expression network analysis was used to find modules of interest and key genes. Functional enrichment analysis was performed to functionally annotate the modules and key genes. The expression levels of key genes in all cancers were validated using Oncomine and Gene Expression Omnibus (GEO) database containing molecular subtypes in BLCA. Protein interaction networks were used to identify upstream genes, and the relationships between genes were analyzed at the protein and transcription levels.

**Findings:** mRNAsi was significantly upregulated in cancer tissues. Corrected mRNAsi in BLCA increased as tumor stage increased, with T3 having the highest stem cell characteristics. Lower corrected mRNAsi scores had better overall survival and treatment outcome. The modules of interest and key genes were determined based on topological overlap measurement clustering results and the inclusion criteria. For 13 key genes (*AURKA, BUB1B, CDCA5, CDCA8, KIF11, KIF18B, KIF2C, KIFC1, KPNA2, NCAPG, NEK2, NUSAP1*, and *RACGAP1*), enriched gene ontology terms related to cell proliferation (e.g., mitotic nuclear division, spindle, and microtubule binding) were determined. Their expression did not differ according to the pathological stages of BLCA, and these genes were clearly overexpressed in many types of cancers. In GEO database, the expression levels of 13 key genes were higher in basal subtype with the highest stem cell characteristics than in luminal and its subtypes. *AURKB* and *PLK1* may be regulated upstream of other key genes, and the key genes were found to be strongly correlated with each other and with upstream genes.

**Interpretation:** The 13 key genes identified in this study were found to play important roles in the maintenance of BLCA stem cells. Controlling the upstream genes *AURKB* and *PLK1* may have applications in the treatment of BLCA. These genes may act as therapeutic targets for inhibiting the stemness characteristics of BLCA.

## Introduction

Bladder cancer (BLCA) is one of the most common cancers worldwide and results in ~150,000 deaths each year. The prognosis of patients with invasive BLCA is still very poor. Approximately 30% of cases of invasive BLCA are associated with occult distant metastasis at the time of diagnosis, leading to a disappointing 5-year survival rate in patients with muscle-invasive BLCA (1).

In recent years, populations of undifferentiated cells with stem cell-like properties in BLCA have been identified as the main factors affecting recurrence and progression (2). Such cancer stemness features have been extensively studied using artificial intelligence and deep learning methods. For example, Tathiane et al. used a one-class logistic regression machine learning algorithm (OCLR) to publish molecular profiles of normal cell types with different degrees of stemness. A dataset of 99 human stem/progenitor cells from the Progenitor Cell Biology Consortium (https://www.synapse.org/pcbc) was profiled using the Illumina HumanMethylation 450 (HM450) platform and used to define stem cell signatures, containing 4 embryonic stem cells (ESCs), 40 induced pluripotent stem cells (iPSCs), 22 stem cell (SC)-derived embryoid bodies (EBs), 11 SC-derived mesoderm (MESO), 11 SC-derived ectoderm (ECTO), and 11 SC-derived definitive endoderm (DE). Stemness indices were derived using an OCLR algorithm trained on stem cell (SC; ESC/iPSC) classes and their differentiated ecto-, meso-, and endoderm progenitors. Additionally, a multiplatform analysis of transcriptomes, methylomes, and transcription factor binding sites was performed to quantify stemness, and a DNA methylation-based stemness index (mDNAsi) and an mRNA expression-based stemness index (mRNAsi) were obtained. Higher mRNAsi scores are associated with active biological processes in cancer stem cells (CSCs) and greater tumor dedifferentiation, as reflected by histopathological grades. These stemness indices have also been applied to datasets from The Cancer Genome Atlas (TCGA) in order to calculate the mRNAsi and mDNAsi scores of the samples (3).

Weighted gene co-expression network analysis (WGCNA), which constructs gene networks in which the connections between gene pairs are identified and weighted based on their associated expression levels (4), has been widely used for processing gene expression data and studying network changes. In short, after processing the expression spectrum into weighted connections, WGCNA can be used to identify network topologies and subnetworks, called modules, using topological overlap dissimilarity as a measure of the distance between genes. Thus, only genes that are highly co-expressed (i.e., related through strongly weighted connections in the network) can constitute a gene module. These modules can be linked to clinical features of interest. The set of genes thus found is biologically more significant than the difference in the amount of expression of the comparative gene (5).

In this study, we aimed to identify key genes associated with stemness by combining WGCNA with BLCA mRNAsi in TCGA. Our results established a novel approach for identifying stemness-related genes and provided insights into the roles of some CSC-related genes in cancer.

## Materials and Methods

### Data Processing

#### Data Download and Pre-processing

The RNA-sequencing (RNA-seq) results of 433 tissues and 408 cases of human bladder transitional cell carcinoma and papilloma samples were obtained from TCGA database (https://portal.gdc.cancer.gov). These data were current as of September 26, 2018. RNA-seq results of 19 normal samples and 414 cancer samples were combined into a matrix file using a merge script in the Perl language (http://www.perl.org/). Next, the Ensembl database (http://asia.ensembl.org/index.html) was used to convert gene names from Ensembl IDs to a matrix of gene symbols. In addition, clinical data from 408 cases were downloaded and filtered for useful information. Because clinical data in TCGA database are continuously updated, the survival time of deceased patients is more accurate than that from other sources.

#### mRNAsi in Molecular Subtypes and Its Clinical Significance

Using TCGA BLCA 412 muscle-invasive bladder cancer (MIBC) samples, comprehensive molecular characterization was performed with APOBEC-related mutational signature (6). Molecular subtyping of each sample was obtained and correlated with mRNAsi. Kruskal–Wallis test were used to determine the significance of differences between subtypes.

To investigate the prognostic value of mRNAsi scores, we performed an overall survival analysis according to mRNAsi scores using GraphPad Prism Mac version 7. Log-rank tests were used to determine statistical significance. After comparing mRNAsi in normal and tumor samples with unpaired *t*-test, the data were combined with pathological staging, and the median mRNAsi score was used to plot a line chart to observe changes in mRNAsi at different stages. Kruskal-Wallis tests were used to determine the significance of differences between groups. Among the data of the treatment results in clinical data, Progressive and Complete Remission were selected to compare the corresponding mRNAsi.

#### Screening of Differentially Expressed Genes (DEGs)

The “edgeR” R package was used to screen expression data for DEGs between normal bladder and cancer samples. The selection criteria were as follows: false discovery rate (FDR) < 0.05, and |log_2_ fold change| > 1. The values for genes with the same names were averaged, and genes whose expression levels were <1 were deleted.

### WGCNA

#### WGCNA and Module Preservation

WGCNA was performed using the WGCNA R package (4). Because some genes with no significant changes in expression between samples were highly correlated in WGCNA, the genes with the most differential expression were used in subsequent WGCNA analyses. Genes with the highest 25% of DEG variance were selected, guaranteeing the heterogeneity, and accuracy of bioinformatics statistics for further co-expression network analysis. First, RNA-seq data were filtered to reduce outliers. The co-expression similarity matrix consisted of the absolute values of the correlation between transcript expression levels. A Pearson correlation matrix was constructed for paired genes. We constructed a weighted adjacency matrix using the power function a_mn_ = |c_mn_|β (c_mn_ = Pearson correlation between gene m and gene n; a_mn_ = adjacency between gene m and gene n). The parameter β emphasized a strong correlation between genes and penalized a weak correlation. Next, an appropriate β value was selected to increase the similarity matrix and achieve a scale-free co-expression network. The adjacency matrix was then converted into a topological overlap matrix (TOM), which measures the network connectivity of genes defined as the sum of adjacent genes generated by all other networks. Average linkage hierarchical clustering was performed based on TOM-based dissimilarity measurements, and the minimum size (genome) of the gene dendrogram was 30. Through further analysis of modules, we calculated their dissimilarity and constructed module dendrograms.

#### Confirmation of Significant Modules

To determine the significance of each module, gene significance (GS) was calculated to measure the correlation between genes and sample traits. Module eigengenes (MEs) are considered to be the main components in the principal component analysis of each gene module, and the expression patterns of all genes can be summarized as a single feature expression profile within a given module. Next, GS was defined as the log_10_ conversion of the *p*-value in the linear regression between gene expression and clinical data (GS = lgP). Module significance (MS) was defined as the average GS within the module and calculated to measure the correlation between the module and sample traits. Statistical significance was determined using the relevant *p*-values. In order to increase the capacity of the modules, we selected a cutoff (<0.25) to merge some modules with similar heights. Here, we selected mRNAsi and epigenetically regulated mRNAsi as the clinical phenotypes (3). The gene modules, combined with the clinical phenotypes, were then analyzed.

#### Key Gene Identification

After selecting modules of interest, we calculated GS and module membership (MM, correlation between the module's own genes and gene expression profiles) for each key gene and set their thresholds. The thresholds for screening key genes in the module were defined as cor. gene MM > 0.8 and cor. gene GS > 0.5.

### Functional Annotation: Gene Ontology (GO) and Kyoto Encyclopedia of Genes and Genomes (KEGG) Analyses

To investigate the biological functions of the module genes and key genes, we used the “clusterProfiler” R package to perform GO functional annotations and KEGG pathway enrichment analysis (7). The threshold values were as follows: *p* < 0.01, and FDR < 0.05.

### Data Validation

Oncomine (http://www.oncomine.org) was used in the microarray database to examine differences in mRNA expression of key genes between tumors and normal tissues in BLCA. The threshold limits were as follows: *p*-value, 1E-4; fold change, 2; gene level, 10%; data type, mRNA. For each key gene, we compared the results for cancerous with those for normal tissues and the results for cancer with those for cancer.

In order to verify the relationship between the expression of key genes and the characteristics of CSCs, we selected GSE87304 and GSE124305 for the molecular division of BLCA in the Gene Expression Omnibus (GEO) database. Because the basal subtype has the highest characteristics of CSCs (6), the differences in the expression of key genes in subtypes such as basal and luminal were further compared. Because CSCs have the characteristics of chemotherapy resistance, we choose GSE52219 to compare the expression differences in key genes. GSE52219 is a study of neoadjuvant chemotherapy in bladder cancer, which contains 17 samples that respond to chemotherapy and 6 that do not respond to chemotherapy (8). The statistical analysis was performed using the *t*-test or Mann-Whitney *U*-test.

### Causal Relationship and Interaction of Proteins

DisNor (https://disnor.uniroma2.it/) was used to generate and investigate a protein interaction network linking disease genes from the causal interaction information annotated in SIGNOR and protein interaction data in Mentha. The Multi-Protein Search module was selected. The first neighbor was the complexity level, which analyzed the causal relationship between proteins. STRING (https://www.string-db.org) version 11.0 was used with the multiple protein modules for protein interaction network analysis.

### Gene Co-expression Analysis

The co-expression relationships between key genes and upstream genes were calculated based on gene expression levels to determine the strength of these relationships at the transcriptional level. The R corrplot package was used to calculate the Pearson correlations between genes.

## Results

### Data Processing

#### mRNAsi in Molecular Subtypes and Clinical Characteristics in BLCA

**Figure 1:**
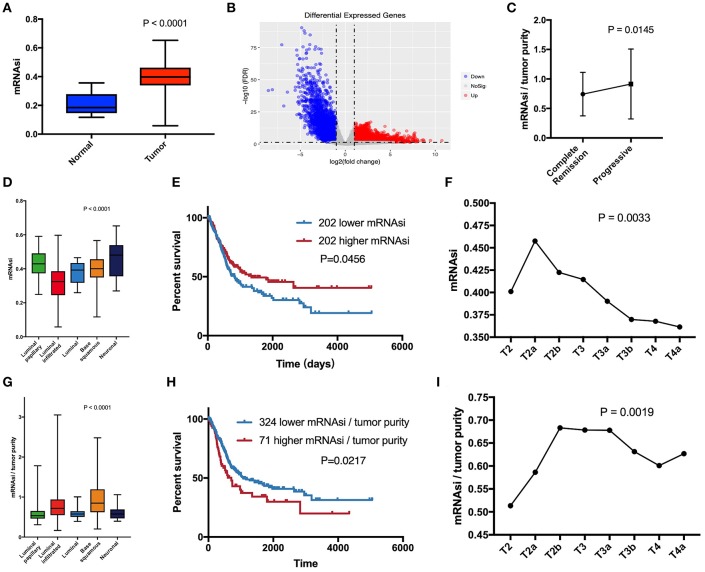
**(A)** Differences in mRNAsi between normal (19 samples) and tumor (404 samples) tissues. **(B)** Differentially expressed genes; blue indicates down-regulated of genes, and red indicates up-regulated of genes. **(C)** After primary treatment, the progression group had a higher corrected mRNAsi (mRNAsi/tumor purity) than the complete remission group. **(D)** Comparison of mRNAsi in five MIBC molecular subtypes. **(E)** Kaplan-Meier curves show that the low mRNAsi group had greater mortality than does the high mRNAsi group. **(F)** Excluding T1 and T4b with small sample size, mRNAsi generally decreases with bladder cancer progression. **(G)** Comparison of corrected mRNAsi in five MIBC molecular subtypes. **(H)** Kaplan–Meier curves show that the higher corrected mRNAsi group had greater mortality than that of the lower mRNAsi group. **(I)** Corrected mRNAsi generally increases from located to advanced.

mRNAsi is an index that describes the degree of similarity between tumor cells and stem cells and can be considered a quantitative representation of CSCs. Obviously, there is a significant difference in mRNAsi between normal and tumor tissues (Figure 1A). Among the five molecular types, there were significant differences in mRNAsi in some subgroups (Figure 1D). The neuronal subtype with the worst prognosis had the highest mRNAsi, but the best prognostic luminal-papillary also had high mRNAsi. This result is different from our understanding of the characteristics of CSCs. In the survival analysis, we observed that patients with higher mRNAsi scores had better overall survival than those with lower mRNAsi scores (Figure 1E). Surprisingly, with the exception of stages T1 (one sample) and T4b (five samples), which had relatively few samples, mRNAsi scores in other tumor stages were at least 10 and showed an overall decreasing trend; the significance of the differences between groups was confirmed using Kruskal-Wallis tests (Figure 1F).

The calculation of mRNAsi was carried out from the molecular profiles of normal cells with varying degrees of stemness (3). Therefore, mRNAsi is a stemness comprehensive score for all cells in a sample, which allowed us to understand that the purity of tumor affected mRNAsi. Absolute tumor purity is derived from the study by Robertson et al. and contains data from 403 patients for subsequent calibration of mRNAsi (6). Finally, we used corrected mRNAsi (mRNAsi/tumor purity) to correct the influence of tumor purity and re-compared the CSC characteristics of the five molecular types (Figure 1G), which yielded results similar to those of the study by Robertson et al. (6). In the data of primary treatment results in clinical data, progressive (178 samples) and complete remission (39 samples) were selected to compare the corresponding corrected mRNAsi (Figure 1C), and the *t*-test was used to verify the significance. The corrected survival analysis and tumor T stage reflected true CSC characteristics in clinical data (Figures 1H,I).

#### Screening of DEGs

Since normal mRNAsi is significantly different from the tumor, we first performed data cleansing to screen out differential genes. Data filtering, normalization, and difference analysis were performed to compare BLCA and normal samples. From this analysis, 8,510 DEGs were screened, of which 5,422 were upregulated, and 3,088 were downregulated (Figure 1B).

### WGCNA: Identification of the Most Significant Modules and Genes

**Figure 2:**
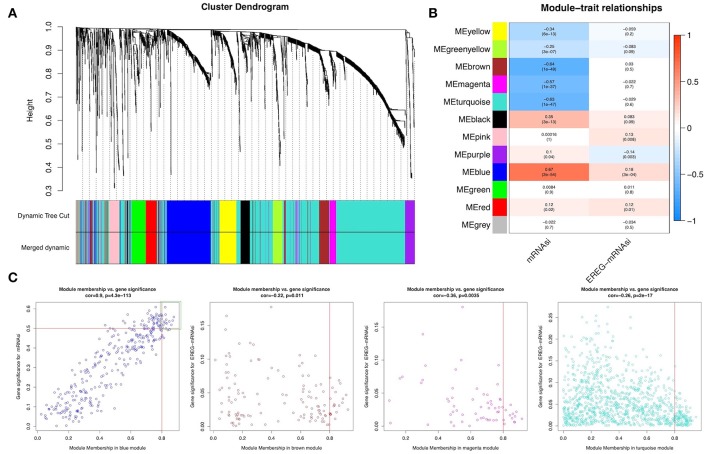
Weighted gene co-expression network of bladder urothelial cancer. **(A)** Identification of a co-expression module in bladder urothelial cancer. The branches of the cluster dendrogram correspond to the 11 different gene modules. Each piece of the leaves on the cluster dendrogram corresponds to a gene. **(B)** Correlation between the gene module and clinical traits, including mRNAsi and EREG-mRNAsi. The correlation coefficient in each cell represented the correlation between the gene module and the clinical traits, which decreased in size from red to blue. The corresponding *P*-value is also annotated. **(C)** Scatter plot of module eigengenes in the blue, brown, magenta, and turquoise modules.

WGCNA was performed to construct a gene co-expression network to identify biologically significant gene modules and to better understand genes associated with BLCA stemness. After outlier samples were eliminated (Supplemental Figure 1A), the 8,510 DEGs with the highest 25% of variance by cluster analysis were placed in a module. In this study, we selected β = 3 (scale-free *R*^2^ = 0.950) as a soft threshold to ensure a scale-free network (Supplemental Figure 1B) and obtained 11 modules for subsequent analysis (Figure 2A).

To analyze the relationships between the modules and stemness indexes of the samples, we used MS as the overall gene expression level of the corresponding module to calculate the correlations with clinical phenotypes. The blue module was most significantly associated with mRNAsi, with a correlation close to 0.7. In addition, the brown, magenta, and turquoise modules exhibited relatively high negative correlations with mRNAsi (Figure 2B). Thus, we chose the blue module as the module of greatest interest and used this module for subsequent analyses.

The threshold for screening key genes in the mRNAsi group was defined as cor. MM > 0.8 and cor. GS > 0.5. We screened 13 key genes [aurora kinase A [*AURKA*], BUB1 mitotic checkpoint serine/threonine kinase B [*BUB1B*], cell division cycle-associated 5 [*CDCA5*], *CDCA8*, kinesin family member 11 [*KIF11*] *KIF18B*, kinesin family member 2C [*KIF2C*], *KIFC1, KPNA2, NCAPG*, NIMA-related kinase 2 [*NEK2*], nucleolar and spindle associated protein 1 [*NUSAP1*], and Rac GTPase-activating protein 1 [*RACGAP1*]], as shown in Figure 2C.

### Functional Annotation of Modules

To elucidate the functional similarities of the module genes, the “clusterProfiler” R package was used for gene enrichment. GO and KEGG analyses showed that the principal biological functions of the blue module were nuclear division, chromosomal region, and ATPase activity, which were mainly involved in the cell cycle and other pathways. The principal functions of the brown, magenta, and turquoise modules were related to the extracellular matrix, endothelial differentiation, and muscle development. The key genes were representative genes of the blue module, and the enriched functions were also related to cell proliferation (Supplemental Figure 2).

### Analysis and Validation of Key Gene Expression

**Figure 3:**
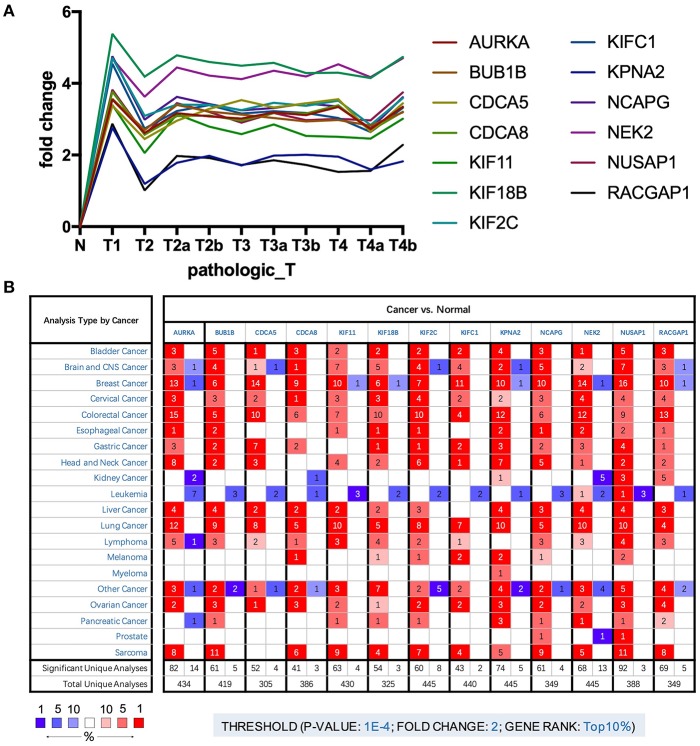
**(A)** Trend of expression of key genes in different stages of bladder cancer. **(B)** The mRNA expression patterns of key genes in overall cancers. The mRNA expression difference between tumors and normal tissues were analyzed in Oncomine database. The number in the colored cell represents the number of analyses meeting these thresholds. The color depth was determined by the gene rank. The red cells indicate that the mRNA levels of target genes are higher in tumor tissues than in normal tissues, while blue cells indicate that the mRNA levels of target genes are lower in tumor tissues than in normal tissues.

To further explore the key genes, we mapped their trends in expression and found that these genes were upregulated in BLCA; however, there were no significant differences between stages (Figure 3A). Through analysis of cancer vs. normal samples using Oncomine, we found that these genes were overexpressed not only in BLCA but also in many other cancers. Twelve genes were ranked among the top 1% in GENE RANK, and 11 genes had two or more data support results (Figure 3B).

**Figure 4:**
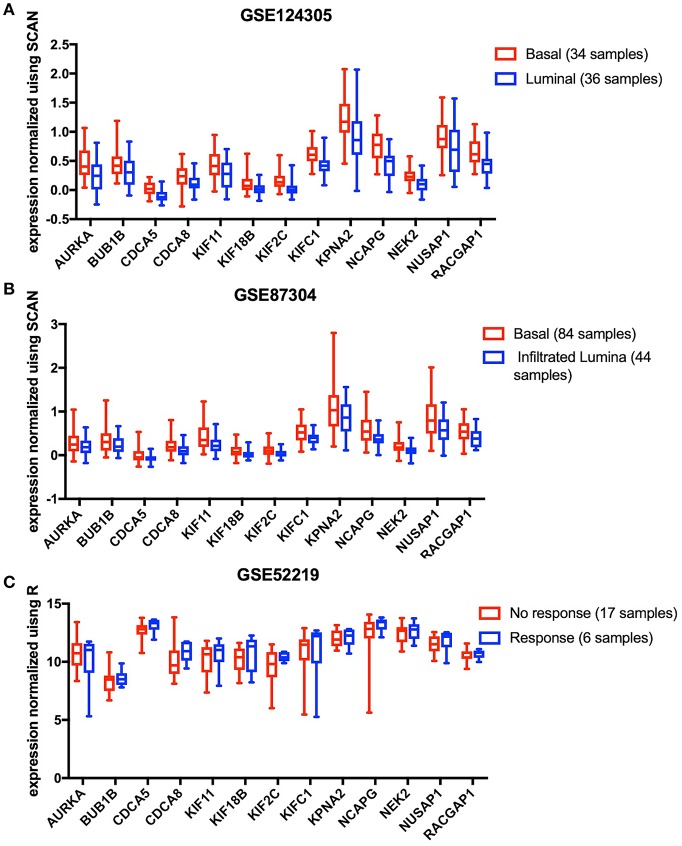
The 13 key genes were verified in the GEO database, and the statistical analysis was performed using the *t*-test or Mann-Whitney *U*-test. **(A)** In GSE124305, expression of the 13 key genes was higher in the basal subtype than the Luminal subtype. **(B)** In GSE87304, expression of the 13 key genes was higher in the basal subtype than the infiltrated Luminal subtype. **(C)** After neoadjuvant chemotherapy, 13 key genes did not differ between the response and no response groups.

The results analyzed in GEO data showed that all the 13 key genes in the basal subtype were more highly expressed (Figures 4A,B), which is also consistent with the result that they maintain CSC characteristics. Although it is known that CSCs exhibit chemotherapy resistance, chemotherapy resistance had been reported to be related to cancer-associated fibroblasts in extracellular matrix (9). In GSE52219, there was no significant difference in the expression of key genes in the groups with chemotherapy response and no response (Figure 4C).

### Causal Relationships and Interactions With Proteins

**Figure 5:**
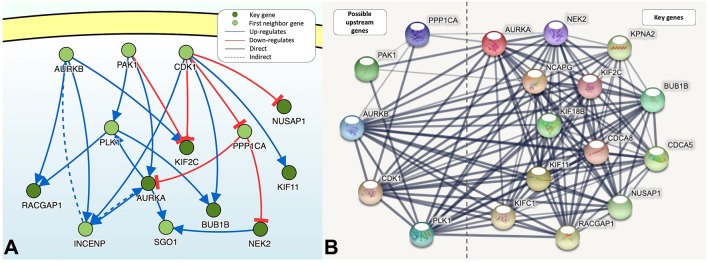
**(A)** The causal interaction of key gene analysis in DisNor. **(B)** The protein-protein interaction between key genes and possible upstream genes. The thickness of the solid line represents the strength of the relationship.

DisNor revealed the first neighbors of seven of the key genes, among which Aurora kinase B (*AURKB*), cyclin-dependent kinase 1 (*CDK1*), p21-activated kinase 1 (*PAK1*), Polo-like kinase 1 (*PLK1*), and protein phosphatase PP1-alpha catalytic subunit alpha (*PPP1CA*) were upstream genes and directly or indirectly affected at least two key genes (Figure 5A). The functions of PAK1 and CDK1 are relatively complex, and both of these proteins can upregulate or downregulate key genes. PPP1CA, which is also upregulated in BLCA, inhibits the expression of key genes. Therefore, *AURKB* and *PLK1* are ideal upstream gene targets. The protein interaction relationships between key genes and upstream genes were validated by STRING, and a broad and strong relationship between the key genes was found (Figure 5B). Among the five upstream genes, the relationships between *PAK1* and *PPP1CA* and the key genes were significantly weaker, whereas the opposite was true for *AURKB* and *PLK1*, further demonstrating that *AURKB* and *PLK1* were ideal upstream target genes.

### Co-expression of Key Genes and Upstream Genes

**Figure 6:**
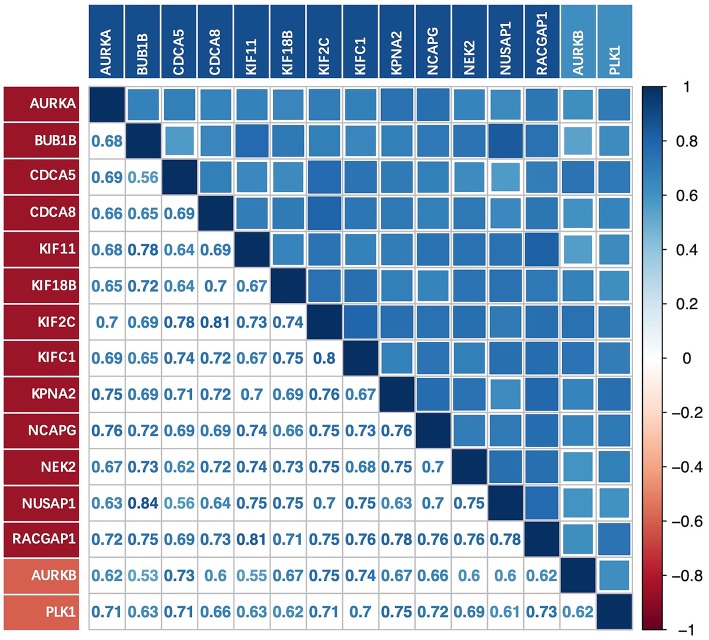
Correlation between key genes and upstream genes AURKB and PLK1 at transcriptional level.

There was a strong correlation between the key genes and the upstream genes *AURKB* and *PLK1* at the transcriptional level (Figure 6); this correlation was statistically significant (*p* < 0.01). The relationship with the lowest correlation was between *BUB1B* and *AURKB* (0.53), whereas that with the highest correlation was between *BUB1B* and *NUSAP1* (0.84). We focused on the upstream genes *AURKB* and *PLK1*. In the experimentally validated protein causal relationship, *AURKB* was highly correlated with *KIF2C, PLK1*, and *RACGAP1*, ranking first and second, respectively. Additionally, the correlation between *AURKB* and *RACGAP1* was relatively low, but >0.6.

## Discussion

BLCA is a complex disease associated with high morbidity and mortality. In recent years, CSCs have been reported to make important contributions to tumor recurrence, progression, and therapeutic resistance (2). Therefore, therapeutic targeting of BLCA stem cells is essential. In this study, we identified key genes associated with CSC characteristics using WGCNA based on mRNAsi scores, as calculated by Tathiane et al. The corrected mRNAsi scores increased as the tumor pathological stage increased, and T3 stage tumors have the highest stem cell characteristics. Higher corrected mRNAsi scores were associated with poorer overall survival and progression after initial treatment in BLCA. The expression levels of key genes significantly upregulated in tumor samples and did not differ significantly among pathological stages. Key genes closely related to pluripotent stem cells have been proven to be over-expressed in most tumors. Moreover, all organ tissues are developed from pluripotent stem cells, suggesting that key genes may play a role in maintaining stem cell properties in a variety of cancers. There was a strong interaction among their proteins, and there was a strong co-expression relationship at the transcriptional level. These results led us to re-evaluate the relationship between CSC characteristics and BLCA progression and to aim to identify upstream genes that may influence cancer development and progression based on key genes.

Undifferentiated primary tumors are more likely to cause cancer cells to spread to distant organs, leading to disease progression and poor prognosis. In addition, CSCs are generally resistant to available therapies (10). The acquisition of progenitor cell-like and stem cell-like characteristics and loss of the differentiated phenotype are manifestations of cancer progression (11), consistent with the increase in BLCA stemness as the tumor progresses. In this study, we focused on changes in progression after tumor development. In our survival analysis, we found that patients with higher corrected mRNAsi scores had lower overall survival rates, which was consistent with the poor prognosis associated with CSC characteristics. T3 stage BLCA had relatively higher CSC characteristics, indicating that stem cell properties begin to rise from initiation phrase of metastasis.

Functional annotations of the blue module were primarily related to stem cell self-renewal and proliferation characteristics. The brown, magenta, and turquoise modules, which were negatively correlated with mRNAsi, were all inversely associated with differentiation and the development of stem cell dedifferentiation characteristics. Next, key genes were selected from the blue module based on GS and MM, and strong protein interaction relationships were found among these genes. In addition, functional enrichment showed that their functions were similar to those of the blue module, highlighting their importance.

Using Oncomine, we found that the upstream genes AURKB, CDK1, PAK1, PLK1, and PPP1CA were significantly overexpressed in BLCA with the exception of PAK1. Through analysis of protein causality and interactions, we believe that AURKB and PLK1 are ideal drug therapeutic targets. First, both of these targets are significantly overexpressed in BLCA and are upstream genes that affect at least two key genes. In addition, similar to key genes, they were identified in the blue module and had a wide range of strong protein interactions with the key genes. However, the functions of PAK1 and CDK1, which are also upstream genes, are relatively complex, and both upregulate and downregulate key genes. PPP1CA, which is overexpressed in BLCA, also inhibits the expression or activity of key genes. The protein interactions between PPP1CA, PAK1, and key genes are also weaker. However, *AURKB, PLK1*, and key genes were strongly co-expressed with respect to transcriptional expression. Thus, we inferred that *AURKB* and *PLK1* were important targets of interest.

The Oncomine results showed that the key genes were overexpressed in a variety of cancers, and 12 of the 13 genes were ranked in the top 1% of Gene Rank for BLCA. The gene sets that maintain the characteristics of stem cells in various cancers may have similarities. Since the formation of various organ tissues occurs from pluripotent stem cells, their CSCs are dedifferentiated with stem cell characteristics. This reverse development has made various CSCs possess some characteristics of pluripotent stem cells. The expression of key genes did not change significantly with tumor progression, indicating that they had maintained the characteristics of stem cells. Moreover, their level of overexpression was related to the level of stemness, and their continued increase may promote changes involved in tumor progression and post-therapy progression. More than half of these genes have been consistently reported in BLCA, and some have also been shown to be associated with the characteristics of CSCs. In studies of the mechanisms of hepatocellular carcinoma metastasis, overexpression of AURKA induces the epithelial-mesenchymal transition and CSC behaviors via the phosphatidylinositol 3-kinase/AKT pathway (12). BUB1B is associated with CSC tumorigenesis and resistance to radiation (13). KIF11 promotes the formation of esophageal squamous cell carcinoma and colorectal cancer cell spheroids; spheroid formation is used to characterize CSCs (14). NEK2 is associated with CSC self-renewal and chemotherapy resistance (15).

The key gene was further validated in GSE87304 and GSE124305, and the expression level was significantly higher in the basal subgroup with the highest CSC characteristics, indicating indirectly that they maintained stem cell characteristics. However, the expression level of the key gene did not differ between the different responses after neoadjuvant chemotherapy, which does not indicate that these genes are not resistant to chemotherapy. Less samples are likely to be indistinguishable and different molecular subtypes of BLCA have different responses to chemotherapy. Cancer-associated fibroblasts also contribute to chemotherapy resistance in the cancer microenvironment (16).

Many studies have shown that CSCs have one or more abnormalities in signaling pathways that regulate self-renewal. The Wnt/β-catenin, Notch, and Hedgehog pathways have been thoroughly studied (17). AURKA, NEK2, and RACGAP1 in the Wnt/β-catenin pathway (18–20) and NUSAP1 in the Hedgehog pathway (21) may be essential for the tumorigenicity of CSCs. These genes are important therapeutic targets for inhibiting the self-renewal, proliferation, and tumor development of CSCs.

The cellular pathways in which the key genes are most involved are pathways associated with extracellular signal-regulated kinase (ERK). In melanoma, FOXM1 mediates AURKA transcriptional activation and expression, and activation of the mitogen-activated protein kinase/ERK pathway drives AURKA expression (22). In many human cancer cells, KIF2C expression positively regulates the signaling pathways downstream of Ras and ERK1/2 (23). In hepatocellular carcinoma, ECT2 enhances the expression and stability of RACGAP1 and accelerates activation of the ECT2-mediated Rho/ERK signaling axis to promote tumor metastasis (24). Silencing of CDCA5 inhibits cell proliferation and induces G_2_/M cell cycle arrest *in vitro*, possibly through inhibition of the ERK/AKT pathway (25). However, decreased BUB1B activity impairs MKP or PP1 activation, leading to elevated levels of MEK and ERK, and feedback models have revealed inhibition of protein phosphatase activity involved in MEK/ERK negative regulation (26).

In conclusion, 13 key genes were found to play important roles in BLCA stem cell maintenance. Controlling the upstream genes *AURKB* and *PLK1* may help in the treatment of BLCA. These genes may be therapeutic targets for inhibiting BLCA stemness characteristics. However, conclusions are based on the retrospective data and hence further biological studies are needed to validate these findings.

## Data Availability

Publicly available datasets were analyzed in this study. This data can be found here: https://portal.gdc.cancer.gov.

## Author Contributions

BL designed the study and drafted the manuscript. BL and SP collected, analyzed, and interpreted the data. YZ, XC, and BW participated in revising the manuscript. All authors have read and approved the final manuscript.

### Conflict of Interest Statement

The authors declare that the research was conducted in the absence of any commercial or financial relationships that could be construed as a potential conflict of interest.

## Supplementary Material

Supplementary Figure 1 **(A)** Clustering of samples and removal of outliers. **(B)** Analysis of network topology for various soft-thresholding powers in scale independence and mean connectivity.

Supplementary Figure 2 GO and KEGG enrichment analyses of modules and key genes of interest. Under a threshold of *p* < 0.01 and FDR < 0.05, the top 10 enriched categories of biological process (BP), cellular component (CC), molecular function (MF), and KEGG pathways are listed.
