# Supplementary figures and images for: Identification of Biomarkers for Controlling Cancer Stem Cell Characteristics in Bladder Cancer by Network Analysis of Transcriptome Data Stemness Indices
Source: Front Oncol. 2019 Jul 4;9:613. doi: 10.3389/fonc.2019.00613 (PMC6620567; doi:10.3389/fonc.2019.00613)

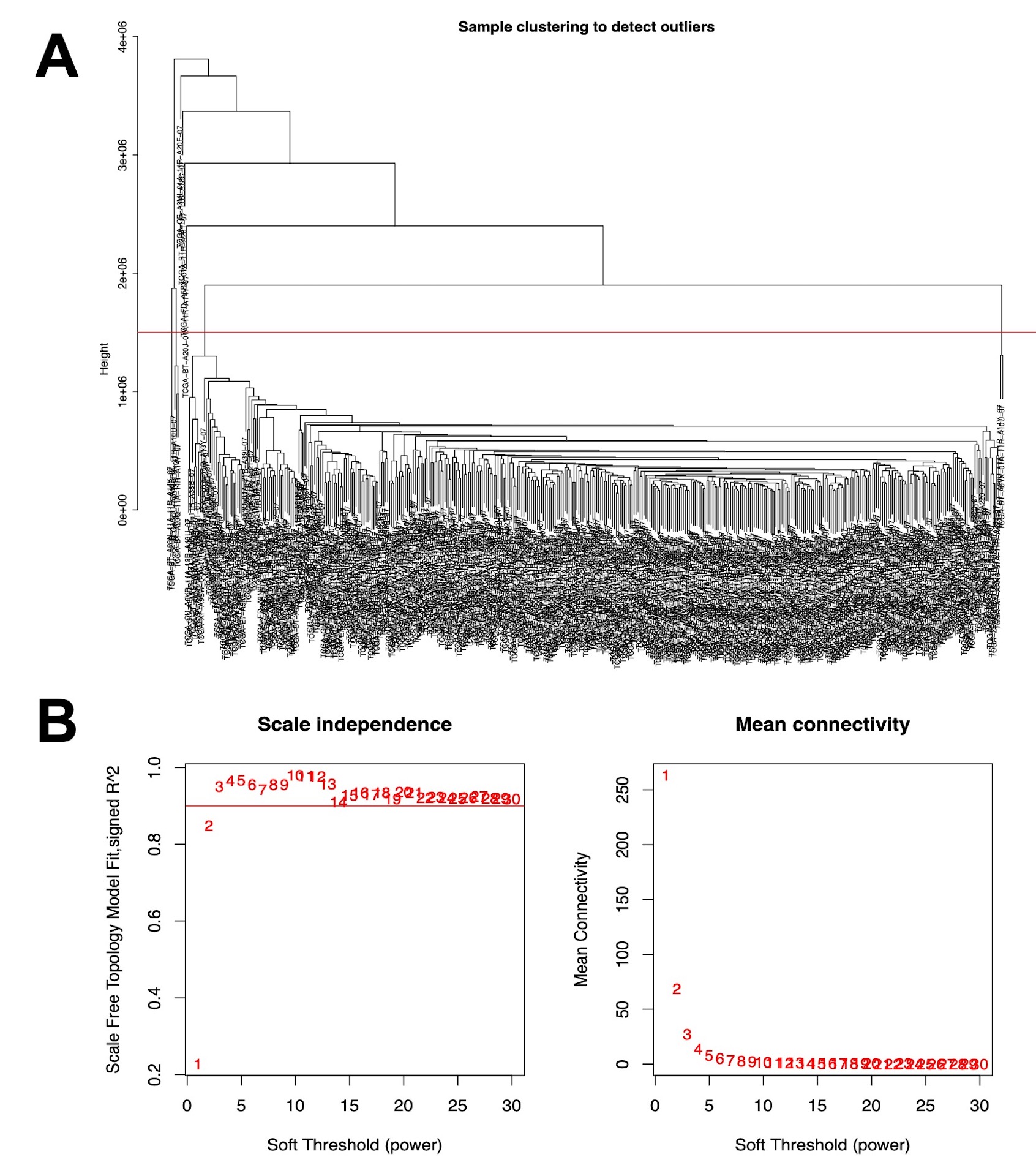

Supplement: Supplementary Figure 1 — (A) Clustering of samples and removal of outliers. (B) Analysis of network topology for various soft-thresholding powers in scale independence and mean connectivity. [file Image_1.JPEG]

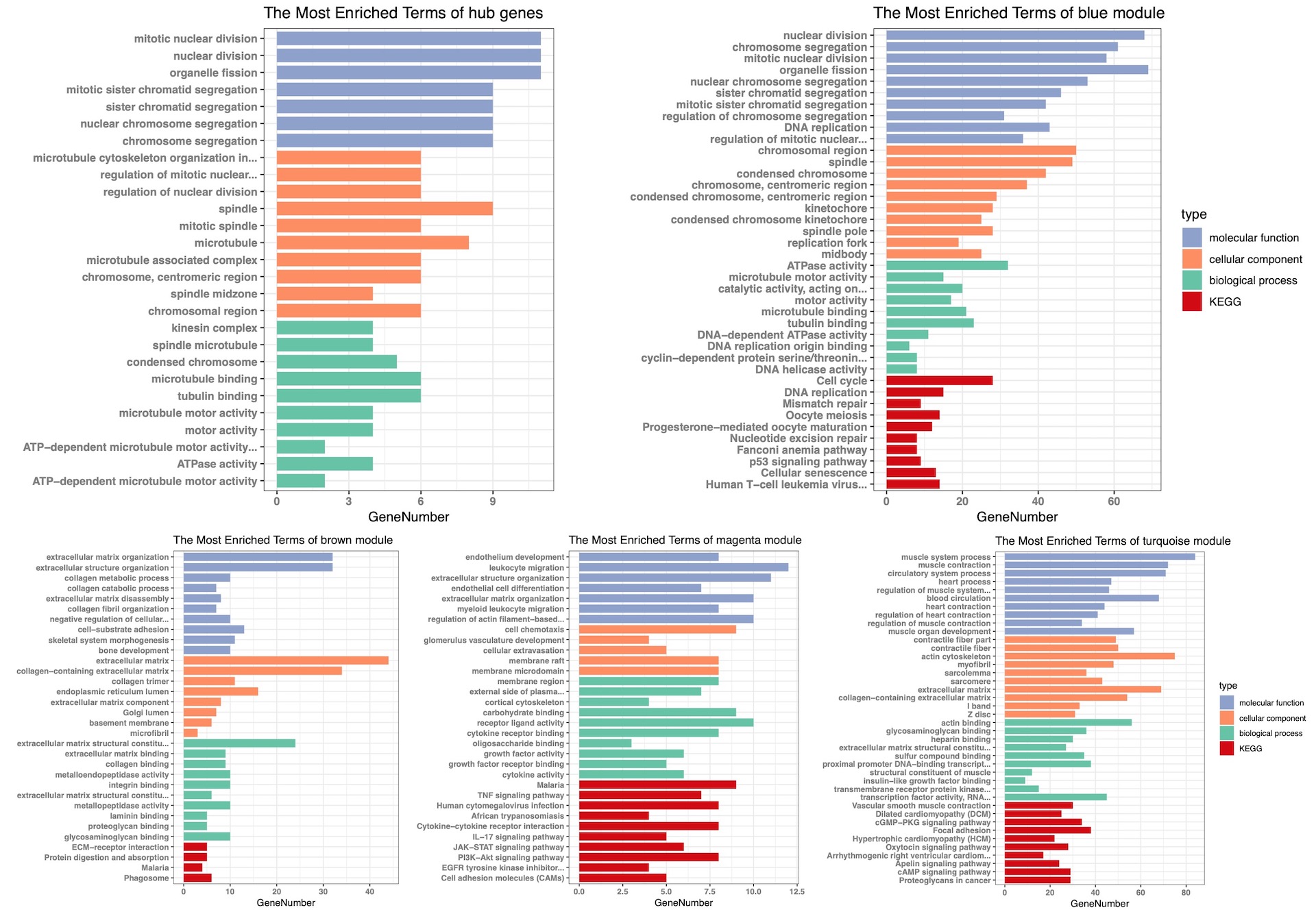

Supplement: Supplementary Figure 2 — GO and KEGG enrichment analyses of modules and key genes of interest. Under a threshold of p < 0.01 and FDR < 0.05, the top 10 enriched categories of biological process (BP), cellular component (CC), molecular function (MF), and KEGG pathways are listed. [file Image_2.JPEG]
